# Whole genome sequence of a non-toxigenic *Corynebacterium diphtheriae* strain from a hospital in southeastern China

**DOI:** 10.1186/s12863-021-00998-9

**Published:** 2021-10-16

**Authors:** Guogang Li, Sipei Wang, Sheng Zhao, Yangxiao Zhou, Xinling Pan

**Affiliations:** 1 Department of Clinical Laboratory, Affiliated Dongyang Hospital of Wenzhou Medical University, Dongyang, Zhejiang, China; 2 Department of Biomedical Sciences Laboratory, Affiliated Dongyang Hospital of Wenzhou Medical University, Dongyang, Zhejiang, China

**Keywords:** *Corynebacterium diphtheriae*, Non-toxigenic, Whole genome sequencing, Belfanti biotype, Virulence factors, Antibiotic resistance, Pathogen-host interaction

## Abstract

**Background:**

Sporadic cases of infection with non-toxigenic *Corynebacterium diphtheriae (C. diphtheriae*) isolates have been reported in regions covered by the Diphtheria-Tetanus-Pertussis vaccine, but no information describing the whole genome of non-toxigenic strains collected in China is available. Therefore, in this work, the complete genome of a non-toxigenic strain of *C. diphtheriae* from a hospital located in southeastern China was performed.

**Results:**

This non-toxigenic isolate belonged to the belfanti biotype and possessed a unique ST (assigned as ST799 in pubMLST). *ErmX* was present in the genome sequence and this isolate owned the resistance to erythromycin and clindamycin. Genes coding for virulence factors involved in adherence, iron-uptake and regulation of diphtheria toxin were also found. Two genes were involved in the interaction between pathogen and host. The phylogenetic analysis revealed that this newly isolated strain was similar to the strain NCTC10838, CMCNS703 and CHUV2995.

**Conclusion:**

Non-toxigenic *C. diphtheriae* strain contained virulence factors, thus it is able to cause an infectious disease, aspect that could be clarified by performing the whole genome sequencing analysis.

**Supplementary Information:**

The online version contains supplementary material available at 10.1186/s12863-021-00998-9.

## Background

Diphtheriae is usually caused by *Coryneabacterium diphtheriae* (*C. diphtheriae*) and it is a potentially lethal disease in children and adults when infected by toxin-producing strains [1]. It spreads among susceptible individuals, resulting in a high mortality in young children without vaccination [2]. Although the vaccine for protection against toxic *C. diphtheriae* has been available for a long time and infants are immunized with a combination of other vaccines such as Diphtheria-Tetanus-Pertussis (DTP) vaccine, sporadic cases or small outbreaks of diphtheriae still occur, especially in regions with low vaccine coverage [3–7].

The reported *C. diphtheriae* isolates are categorized as toxigenic and non-toxigenic according to the presence of the diphtheria toxin. The infection cases caused by the toxigenic strains declined after vaccine immunization program, but the current vaccines may not protect susceptible individuals from the non-toxigenic strains, which can also cause severe disease [8, 9]. Thus, the non-toxigenic strains with invasive ability including nontoxigenic but toxin-gene bearing strains should not be ignored [10]. The worst aspect is that the non-toxigenic strains may change to the toxigenic ones through lysogenic conversion [10]. Therefore, routine surveillance of both the toxigenic and non-toxigenic strains of *C. diphtheriae* is necessary to prevent potential outbreaks. There were four biotypes (mitis, gravis, intermedius and belfanti) in clinical *C. diphtheriae* isolates, but the belfanti biotype seemed to be rarely reported and appeared later than other biotypes [11].

The molecular genotyping of *C. diphtheriae* isolates is a useful approach to monitor the transmission or the original isolate during the outbreaks of infectious diseases. Multiple locus sequence typing based on seven housekeeping genes are generally used in *C. diphtheriae* studies. However, routine genotyping is not enough to evaluate its pathogenicity or possibility to infect host and transmission among individuals. Whole genome sequencing has become more suitable in the investigation of non-toxigenic *C. diphtheriae* isolates collected in regions covered by the DTP vaccine.

In this study, a non-toxigenic *C. diphtheriae* strain was collected from the bronchial alveolar lavage fluid collected from a patient aged 57 years [12] who showed symptoms including cough, expectoration and fever at diagnosis. Although non-toxigenic isolates were also reported in China, no information describing the whole genome is available [12–14]. Therefore, in this work, the complete genome of *C. diphtheriae* strain was sequenced, which could help researcher to understand the potential pathogenesis of a non-toxigenic strain.

## Results

### Whole genome assembly and gene annotation

The isolate contained a circular genome of 2,960,956 bp and a linear plasmid of 35,314 bp. According to the blast results, the linear plasmid showed a sequence identity greater than 99% compared to two *C. diphtheriae* strains (ChUV2995) and subspecies lausannense strain (CMCNS703). The strains C. sp. NML93–0612 possessed a sequence identity greater than 90% to our strain, but its coverages was 56%. Other strains showed less than 30% coverage (data not shown).

A total of 3108 and 11 pseudogenes were annotated. The characteristic of CRISPR was shown as number of spacers from CRISPR 1 to CRISPR 9: 1–1–1-11–1-2-6-2-1. A total of 79 non-coding RNAs were predicted from the complete genome, and included 15 rRNA, 53 tRNA and 11 other non-coding RNAs.

### Identification of species and MLST

The *C. diphtheriae* strain was identified as *C. diphtheriae* biotype belfanti through the use of rMLST, with a 97% support. This isolate turned out to be a new type when analyzed by 7 housekeeping genes for determining the MLST type, nearest to ST612 and ST35 in the database. The detailed information for each locus is shown in Table 1. The locus *atpA*, *leuA* and *rpoB* in this study possessed mutations compared to the isolates in the database, when the remaining loci matched exactly to the alleles. The new mutation at locus *atpA*, *leuA* and *rpoB* had been submitted to pubMLST database and this new MLST type was assigned as ST799.

**Table 1:** Multiple loci sequence type analysis of isolate in this study

Locus	This study	ST612	ST35
*atpA*	**66**	6	6
*dnaE*	7	7	7
*dnaK*	21	21	21
*fusA*	12	12	12
*leuA*	**101**	9	15
*odhA*	7	7	7
*rpoB*	**70**	11	11

### Resistance gene and phenotype of the collected *C. diphtheriae*

The complete genome analysis revealed that one gene conferring drug resistance (*ErmX*) coding an rRNA methyltransferase was found. The susceptibility to erythromycin and clincamycin was determined by disk diffusion method. We found this isolated *C. diphtheriae* was both resistant to erythromycin and clindamycin (supplementary Fig. 1).

### Prediction of virulence factors

The gene encoding the diphtheria toxin was not found in this isolate, but the regulation *dtxR* gene existed. In addition, genes involved in adherence, iron uptake, and regulation of diphtheria toxin were also found in the genome (Table 2). In detail, two genes (*srtB* for encoding SpaD-type pili and *sapD* for encoding surface-anchored pilus protein, respectively) were present in genome. Moreover, more copy numbers of genes involved in ABC transporter were also found compared to *C. diphtheriae* NCTC 13129.

**Table 2:** Virulence factors predicted in this non-toxigenic *C. diphtheriae* isolate

class	Virulence factors	Related genes	*C. diphtheriae* in this study	*C. diphtheriae* NCTC 13129 (biotype gravis)
Adherence	SpaD-type pili	srtB	GE000724	DIP0233
	Surface-anchored pilus proteins	sapD	GE000470	DIP0443
Iron uptake	ABC transporter	fagA	GE000031; GE001029; GE001042; GE002284; GE003092	DIP1061
		fagB	GE000032; GE001030; GE002283; GE003093	DIP1060
		fagC	GE000033; GE001031; GE001044; GE002282	DIP1059
		fagD	GE000030; GE001032; GE002285; GE003091	DIP1062
	ABC-type heme transporter	hmuT	GE001688	DIP0626
		hmuU	GE001689	DIP0627
		hmuV	GE001690	DIP0628
	Ciu iron uptake and siderophore biosynthesis system	ciuA	GE001639	DIP0582
		ciuB	GE001640	DIP0583
		ciuC	GE001641	DIP0584
		ciuD	GE001642	DIP0585
		ciuE	GE001643	DIP0586
	Siderophore-dependent iron uptake system	irp6A	GE000857	DIP0108
		irp6B	GE000856	DIP0109
		irp6C	GE000855	DIP0110
Regulation	Diphtheria toxin repressor DtxR	dtxR	GE002692	DIP1414
	Sigma A (Mycobacterium)	sigA/rpoV	GE002685	–
	Sigma H (Mycobacterium)	sigH	GE000444	–

According to the results of the PHI database, two potential virulence factors were predicted, which were not in the database of the virulence factors. The sequence of GE1800 possessed a sequence identity of 99.4% with DIP0733 in the *C. diphtheriae* strain NCTC 13129. In addition, another gene such as GE2120 shared an identity of 95.5% with GE0813 in the strain CDCE8392.

### Phylogenetic analysis based on the whole genome and housekeeping genes

**Fig. 1:**
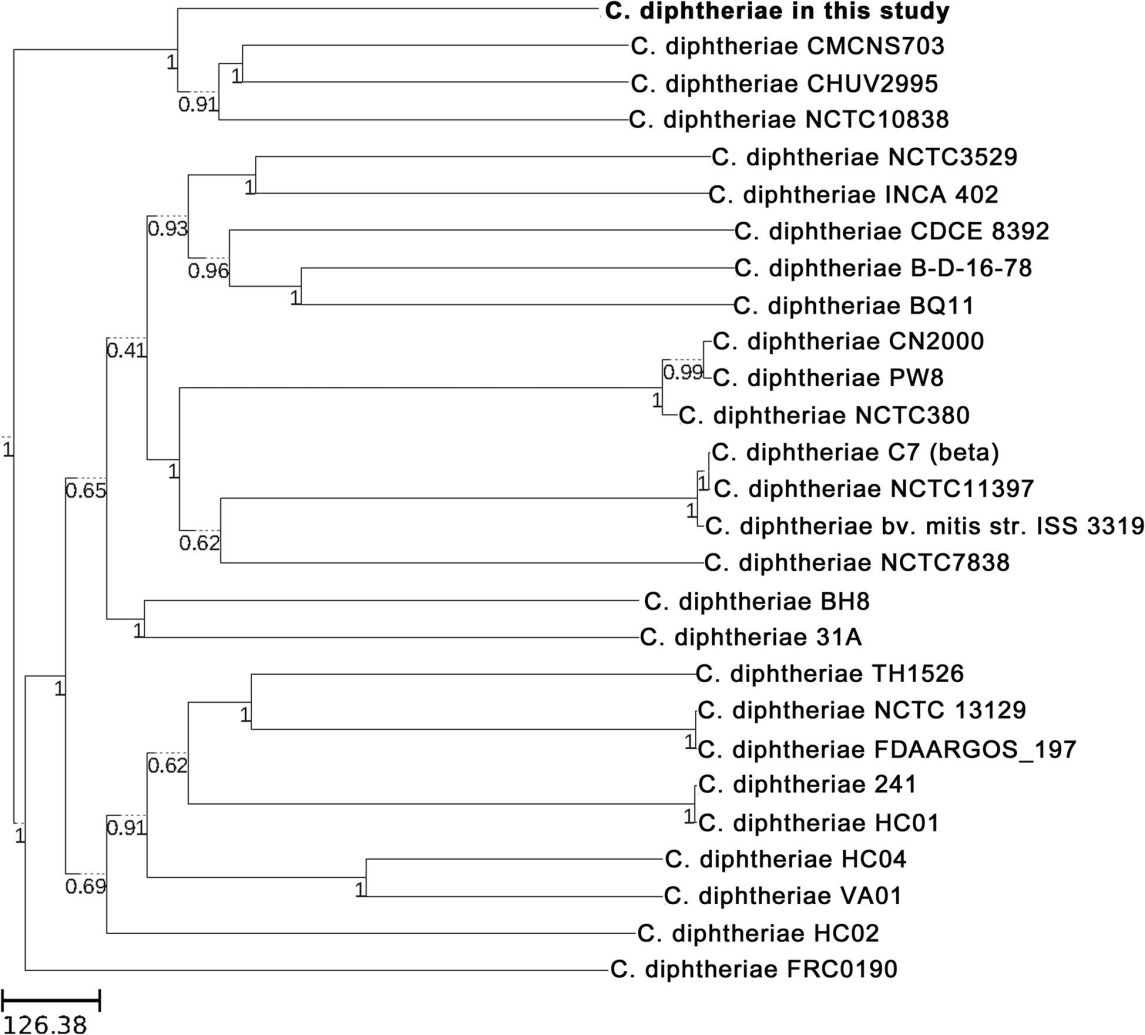
The wgMLST tree based on genomes from database and this *C. diphtheriae* isolate

A total of 26 isolates with whole genome sequences were downloaded from NCBI to compare the similarity between the published *C. diphtheriae* strains and the isolate strain in this region (supplementary Table 1). Twenty-seven whole genome sequences were analyzed including the strain collected in our hospital and the results showed that 1519 genes belonged to the core genes. Then, the wgMLST tree was performed according to these core genes (Fig. 1). The *C. diphtheriae* isolate collected in this study was more similar to the strain NCTC10838 (Australia, throat swab, biotype belfanti), CMCNS703 (India, nasal swab) and CHUV2995 (Switzerland, broncho-alveolar lavage, biotype mitis or belfanti) than other isolates.

**Fig. 2:**
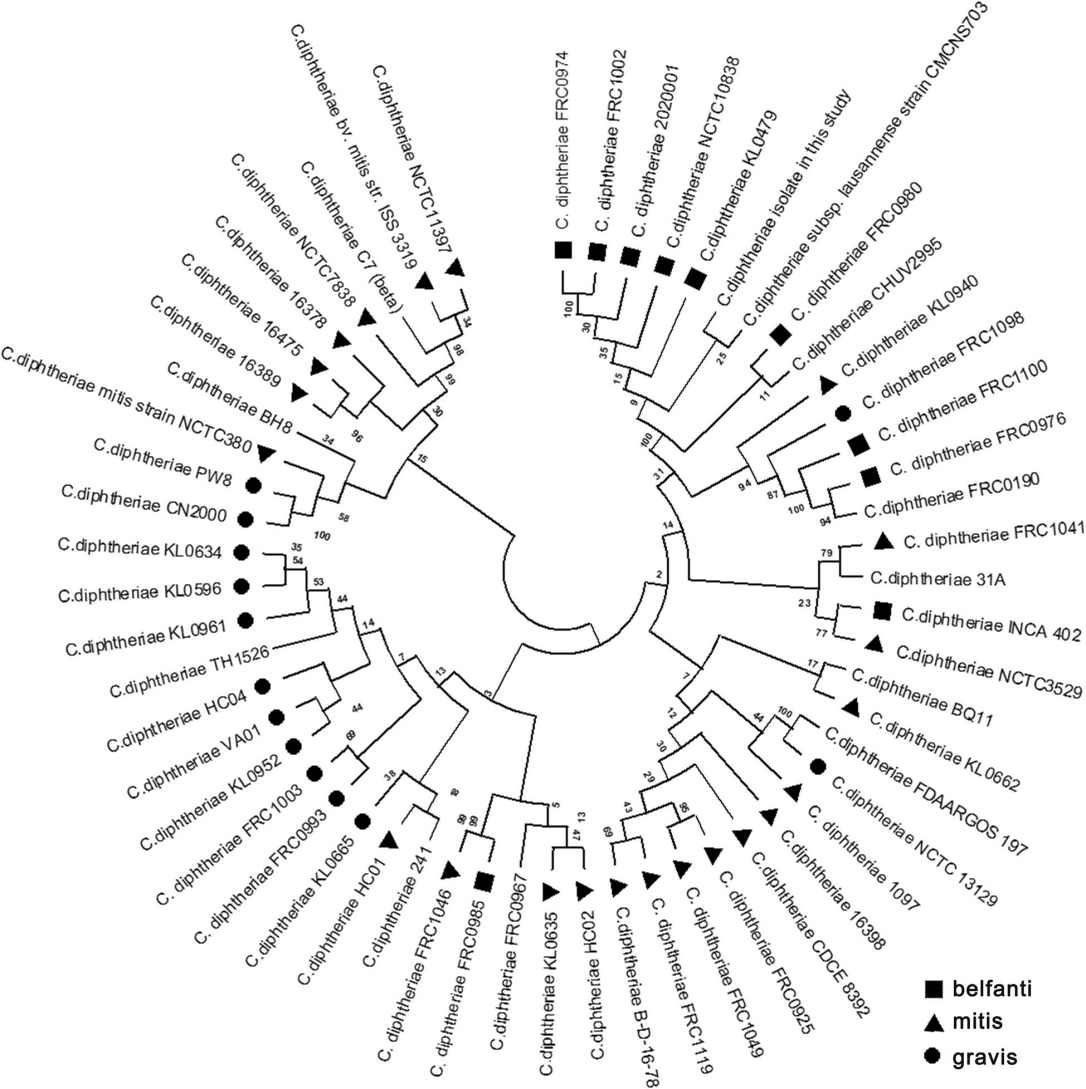
The evolutionary phylogenetic tree of 57 *C. diphtheriae* isolates based on 7 house-keeping genes

A total of 57 *C. diphtheriae* were collected to extract the sequences from seven housekeeping genes and the evolutionary phylogenetic tree was constructed based on them (Fig. 2). The *C. diphtheriae* isolate collected in this study was distributed closer to the strains NCTC10838, CMCNS703, CHUV2995 and KL0479.

## Discussion

One non-toxigenic *C. diphtheriae* was collected in this study and identified as *C. diphtheriae belfanti* according to the complete genome sequence. MLST analysis revealed this new sequence type and potential virulence factors were also predicted in this genome.

The *C. diphtheriae* isolate collected in this study was identified as the belfanti biotype, which is usually considered as non-toxigenic and proposed with the name *C. belfanti* [15]. The patient in this study did not show pseudo-membrane, but had symptoms related to an infection of *C. diphtheriae* including cough, fever and expectoration accompanied with ozena. A study from France revealed that *C. belfanti* can colonize susceptible individuals such as patients with cystic fibrosis, who can infect each other [16]. In addition, *C. belfanti* isolates from Algeria are phylogenetically grouped and associated with ozena, indicating that the infection site and symptoms may be specific for *C. belfanti* [17].

Whole genome sequencing and MLST analysis of isolated strains was essential in investigating the molecular prevalence of pathogens. Sharing the same ST type and core genes among isolates from temporospatial related patients indicated the potential ability of transmission of the non-toxigenic strains. However, this *C. diphtheriae* strain had unique ST (ST799) with mutations in *atpA*, *leuA* and *rpoB, *whichwas more similar to the ST612 and ST35 according to the published data. However, evidence regarding transmission events related to this isolate was not found during the follow-up [12].

Although the diphtheriae toxin was not found in the isolated strain, its regulatory gene *dtxR* was present. Once integrated into specific sites by the tox-encoding bacteriophage, the non-toxigenic strain might be converted into the toxigenic isolate in theory [10]. Among the virulence factors, genes involved in adherence, iron uptake and regulation of diphtheria toxin were also found in this non-toxigenic strain. The pili were essential for bacteria to adhere the epithelial cells and there were genes coding for different types of pili in the genome of *C. diphtheriae*. The spaA-type pili were prevalent in clinical isolates, but the genes for spaD or spaH-type pili were heterogenous as described in previous study [18]. In this isolate, only one gene (*srtB*) for spaD-type pili were found, indicating that the genes for spaABC-type pili might be absent in some non-toxigenic isolates [19, 20]. Moreover, more copies of genes involved in the ABC transporter were present in this isolate compared to the reference genome (NCTC 13129), suggesting its potential increase in the ability to uptake iron and nutrition [21, 22].

Two genes potentially involved in the interaction between host and pathogens were found in this study. DIP0733 (GE1800 in this isolate) could contribute to the binding of *C. diphtheriae* to the proteins of the extracellular matrix, thus potentially contributing its escape in immune response [23]. In addition, the DIP0733 protein could increase its ability to invade epithelial cell, as revealed by experiments in an animal model [23, 24]. The ability of *C. diphtheriae* to interact with epithelial cell is mainly dependent on the C-terminal coiled-coil domain structure of DIP0733, since mutant type strains showed a decreased virulence to invertebrate animals [25]. The C-terminal sequence of GE1800 in this study was completely identical to that of DIP0733, suggesting its potential ability of infection and consequent pathogenesis. Another gene GE2120, which was homologous to GE0813 in the strain CDCE8392, was involved in tellurite resistance. The presence of the GE0813 gene not only enhances the survival of pathogens in the natural environment, but increases the lethality of *Caenorhabditis elegans* and its survival inside human epithelial cells [26].

A gene encoding rRNA methyltransferase (*ErmX*) was found in the genome. ErmX can protect the ribosomes from inactivation because it binds to the antibiotics, and it was indeed involved in the resistance to macrolide, lincosamide and streptogramin. Previous studies reported that *C. diphtheriae* carrying *ErmX* is closely related to the resistance to macrolide, and the *ErmX* is the most common gene in macrolide-resistance corynebacterial strains [27–29], which was supported by the fact this isolate was resistant to erythromycin and clindamycin in this study.

## Conclusions

Non-toxigenic *C. diphtheriae* strains could be pathogenic and cause sporadic disease. Thus, the analysis of the whole genome sequence could help the understanding of the molecular mechanism associated to the pathogenesis of the diseases.

## Methods

### Strain isolation and species identification

The *C. diphtheriae* was collected from the bronchial alveolar lavage fluid collected from a patient aged 57 years who had cough, expectoration, fever and white debris in the larynx at diagnosis. The sample was cultured on a blood agar plate and incubated at 35 °C under 5% CO_2_ for 24 h. At the end of the incubation time, white colony formed and was analyzed for species identification using IVD model by Matrix-Assisted Laser Desorption/Ionization Time of Flight Mass Spectrometry (VITEK, German).

### Genome sequencing and assembly

The bacterium was collected from the blood agar plate, placed in an Eppendorf tube and stored in liquid nitrogen. The genome was extracted using QIAGEN Genomic-tip according to the manufacturer’s instructions (QIAGEN, German). The sequencing data was generated ONT PromethION by LC-Bio [30]. The reads were assembled into sequence by using Canu v1.5 / wtdbg v2.2 software as described previously [31, 32]. The genome sequence was available on NCBI (CP074413).

### Determination of multiple loci sequence type

Species identification based on genome was performed using Ribosomal Multi-locus Sequence Typing (rMLST, https://pubmlst.org/species-id) as previously described [33, 34]. Sequence type based on *atpA*, *dnaE*, *dnaK*, *fusA*, *leuA*, *odhA*, and *rpoB* was analyzed in PubMLST (https://pubmlst.org/organisms/corynebacterium-diphtheriae) [35].

### Phylogenetic tree construction based on core genes and housekeeping genes

Whole genome sequences were uploaded into PGAdg-builder (http://wgmlstdb.imst.nsysu.edu.tw/) [36] and a scheme consisting of core genes was established with a cut off value of the occurrence percentage of more than 95%. Then, the wgMLSTtree was established based on the core genes with default parameters (90% coverage and 90% identity).

A combination of 26 genome sequences mentioned above and 30 *C. diphtheriae* sequences from pubMLST database were analyzed to extract the sequences of seven housekeeping genes (updated by 4th Feb, 2021) to obtain a sequence of 2544 bp length consisting of fragments from *atpA* (378 bp), *dnaE* (354 bp), *dnaK* (345 bp), *fusA* (360 bp), *leuA* (384 bp), *odhA* (381 bp) and *rpoB* (342 bp). Then, the alignment of the sequences was constructed by clustaW in Mega X. The evolutionary history was analyzed using the Maximum Likelihood method and Tamura-Nei model in Mega X [37]. The bootstrap consensus tree performed from 1000 replicates [38] was used to represent the evolutionary history of the analyzed taxa [3]. Branches corresponding to partitions reproduced in less than 50% bootstrap replicates were collapsed. The initial tree(s) for the heuristic search were automatically obtained by applying the Neighbor-Join and BioNJ algorithms to a matrix of pairwise distances estimated using the Tamura-Nei model, and then by selecting the topology with a superior log likelihood value.

### Virulence factors analysis

The whole sequence with the annotated coding sequence was uploaded to the virulence factor database (VFDB, http://www.mgc.ac.cn/VFs/) and analyzed using VFanalyzer [39]. The *C. diphtheriae* NCTC 13129 was the reference genome used as comparison.

### Drug resistance gene and phenotype determination

The assembled genome sequence was uploaded and analyzed using The Comprehensive Antibiotic Resistance Database (https://card.mcmaster.ca/) [40]. The potential gene conferring drug resistance in all bacteria was predicted by the protein homolog model.

The phenotype of antibiotic resistance was determined by disk diffusion method proposed by The European Committee on Antimicrobial susceptibility Testing (https://www.eucast.org/ast_of_bacteria/). In brief, 0.5 McFarland of bacterium was smeared on the blood culture plate. A 6 mm filter paper disk with 2 μg of clindamycin (OXOID, England) or 15 μg of erythromycin (CONT, China) was plated on the culture plates and incubated at 35 °C for 24 h. The inhibition zone diameters were obtained and phenotype was determined based on the breakpoints [41] (https://www.eucast.org/clinical_breakpoints/).

## Supplementary Information

**Additional file 1 Supplementary Fig. 1** The inhibition zone diameters of tested antibiotics. (A) erythromycin; (B) clindamycin.

**Additional file 2. **The accession number information of 26 whole genome sequences involved in this study.

## Data Availability

All data generated or analyzed during this study are included in this published article. The whole genome sequence of newly isolated *Corynebacterium diphtheriae* was uploaded in NCBI with accession number of CP074413.

## Abbreviations

MLST: multi-locus sequence typing
