# Supplementary figures and images for: Whole genome sequence of a non-toxigenic Corynebacterium diphtheriae strain from a hospital in southeastern China
Source: BMC Genom Data. 2021 Oct 16;22:42. doi: 10.1186/s12863-021-00998-9 (PMC8520229; doi:10.1186/s12863-021-00998-9)

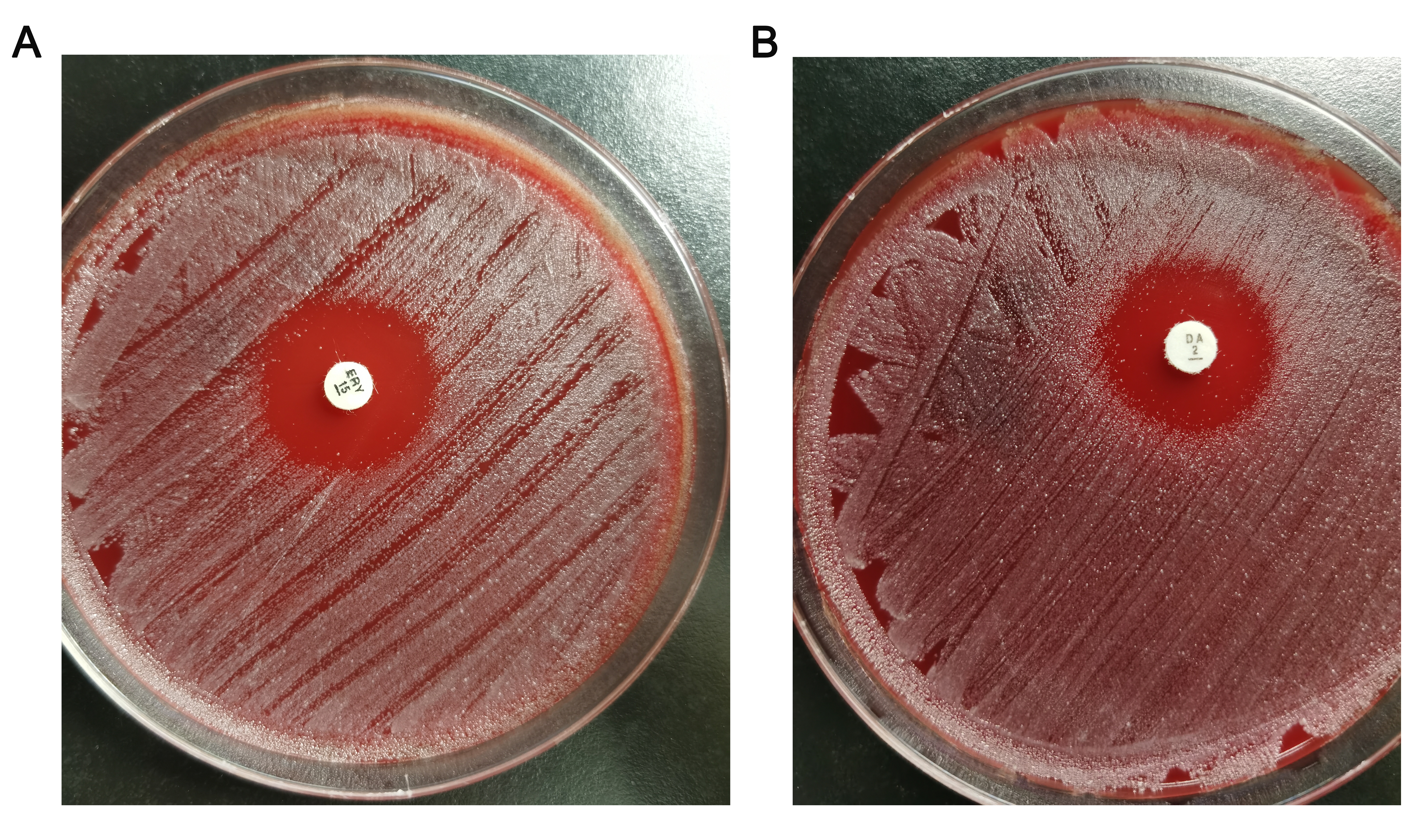

Supplement: Supplementary file 1 — Additional file 1 Supplementary Fig. 1 The inhibition zone diameters of tested antibiotics. (A) erythromycin; (B) clindamycin. [file 12863_2021_998_MOESM1_ESM.tif]
