# Supplementary material for: Whole genome sequence of a non-toxigenic Corynebacterium diphtheriae strain from a hospital in southeastern China
Source: BMC Genom Data. 2021 Oct 16;22:42. doi: 10.1186/s12863-021-00998-9 (PMC8520229; doi:10.1186/s12863-021-00998-9)
Supplement: Supplementary file 2 — Additional file 2. The accession number information of 26 whole genome sequences involved in this study. [file 12863_2021_998_MOESM2_ESM.docx]

| Sequence access number | Isolate name | Biotype |
| --- | --- | --- |
| NC_016782.1 | 241 | - |
| NC_016783.1 | INCA 402 | belfanti |
| NC_016785.1 | CDCE 8392 | mitis |
| NC_002935.2 | NCTC 13129 | gravis |
| NC_016786.1 | HC01 | mitis |
| NC_016788.1 | HC04 | gravis |
| NC_016789.1 | PW8 | gravis |
| NC_016790.1 | VA01 | gravis |
| CP018331.1 | B-D-16-78 | mitis |
| CP020410.2 | FDAARGOS_197 |  |
| NC_016801.1 | C7 (beta) | - |
| NC_016802.1 | HC02 | mitis |
| NC_016800.1 | BH8 | - |
| NC_016799.1 | 31A | - |
| CP025209.1 | bv. mitis str. ISS 3319 | mitis |
| CP029644.1 | BQ11 | - |
| LN831026.1 | NCTC11397 | mitis |
| LR134537.1 | NCTC7838 | mitis |
| LR134538.1 | NCTC3529 | mitis |
| CP038504.1 | TH1526 | - |
| CP038789.1 | subsp. lausannense strain CMCNS703 | - |
| UFWY01000001.1 | NCTC10838 | belfanti |
| CP039522.1 | CN2000 | gravis |
| NZ_UFXN01000001.1 | NCTC380 | mitis |
| LT990688.1 | CHUV2995 | mitis or belfanti |
| LR738855.1 | FRC0190 | - |

Supplementary table. The accession number of whole genome sequence involved this study.

-, the biotype information was not available from published data.
